# Reduced Prefrontal Activation During the Tower of London and Verbal Fluency Task in Patients With Bipolar Depression: A Multi-Channel NIRS Study

**DOI:** 10.3389/fpsyt.2018.00214

**Published:** 2018-05-28

**Authors:** Linyan Fu, Dan Xiang, Jiawei Xiao, Lihua Yao, Ying Wang, Ling Xiao, Huiling Wang, Gaohua Wang, Zhongchun Liu

**Affiliations:** ^1^Department of Psychiatry, Renmin Hospital of Wuhan University, Wuhan, China; ^2^Institute of Neuropsychiatry, Renmin Hospital, Wuhan University, Wuhan, China

**Keywords:** bipolar depression, near-infrared spectroscopy, executive function, the verbal fluency task, the Tower of London task

## Abstract

**Background:** The Tower of London (TOL) task is one of the most commonly used tests for evaluating executive functions, and can indicate planning and problem-solving abilities. The aim of this study was to evaluate hemodynamic changes between the task period and rest period in patients with bipolar depression during the TOL task and the verbal fluency task (VFT) using near-infrared spectroscopy (NIRS).

**Methods:** Forty-three patients with bipolar depression and 32 healthy controls (HCs) matched for sex, age, handedness, and years of education were enrolled in this study. All participants were aged between 16 and 50. All patients in our study were taking medications such as antidepressants, antipsychotics and mood stabilizers at the time of measurement. Changes in oxygenated hemoglobin (oxy-Hb) levels in frontal areas during the TOL task and VFT were evaluated using a 41-channel NIRS system.

**Results:** During the TOL task, the patients with bipolar depression exhibited significantly smaller changes in the bilateral dorsal-lateral prefrontal cortex (DLPFC) than the HCs. During the VFT task, the patients with bipolar depression exhibited significantly smaller changes in the right ventrolateral prefrontal cortex (VLPFC), the right DLPFC and both the right and left prefrontal cortex (PFC) than the HCs.

**Limitations:** Our sample size was small, and the effects of medication cannot be excluded.

**Conclusions:** These results indicate that planning and problem solving dysfunction is related to the impairment of the prefrontal cortex in patients with bipolar depression, and NIRS can be used to assess planning and problem solving abilities, which are essential to daily life in patients with bipolar disorder.

## Introduction

Bipolar disorder is a major psychiatric disorder that is characterized by moods that alternate between episodes of depression and mania or hypomania. Because this disease lacks objective and definitive biomarkers and its pathological and pathophysiological mechanisms are still unclear (1), as with other psychiatric disorders, the diagnosis of bipolar disorder depends on clinical conversations using a diagnostic system such as the International Classification of Diseases (ICD) (2, 3). Bipolar disorder is also associated with high mortality and morbidity (1, 4). Previous studies have shown that both the acute and euthymic bipolar patients present cognitive impairments, which had a negative correlation with quality of life (5, 6).

Many neuroimaging studies using positron emission tomography (PET) and functional magnetic resonance image (fMRI) have demonstrated structural and functional abnormalities in different brain regions in patients with schizophrenia, bipolar disorder and major depressive disorder (MDD) (7, 8). Previous studies have suggested that patients with bipolar disorder have abnormal activation in the frontal and temporal regions, which are known to be related to attention and executive function (9, 10). With the attenuation of symptoms, deficits in those functions can improve (11).

Multichannel near-infrared spectroscopy (NIRS) is a recently developed functional neuroimaging technology that can detect oxygenated hemoglobin concentrations (oxy-Hb) and deoxygenated hemoglobin concentrations (deoxy-Hb) in the brain cortex. Compared with other neuroimaging techniques, NIRS has the following advantages: it is completely non-invasive and has a low cost; it is insensitive to artificial motion; it can capture high-temporal-resolution (0.1 s) changes in hemodynamic concentrations (12); and participants can sit comfortably during the test. In addition, the operation of NIRS does not require radiographers and free from radiation; NIRS is compatible with a variety of neuroimaging instruments (13) and some NIRS devices are portable (14). In Japan, NIRS has been approved by the Ministry of Health, Labor and Welfare for use as a medical technology for the diagnosis of psychiatric disorders (15). Many NIRS studies using verbal fluency task (VFT) to assess cognitive function in the prefrontal cortex and temporal cortex have reported decreased activation in many psychiatric disorders such as major depression (16), bipolar disorder (17), and schizophrenia (18) compared with normal controls. However, VFT only covers a restricted aspect of executive functioning, which would be inadequate in delineating and differentiating complex psychiatric disorder including bipolar disorder (19).

The Tower of London (TOL) task is a classical experiment for evaluating executive functions; unlike VFT, which emphasize information processing and memorizing ability, the TOL task mainly reflects planning and problem-solving abilities. As one of the most commonly used problem-solving tests, it has been widely used for clinical applications and in research since it was created to assess planning and problem-solving ability in patients with damage in various brain regions (20). To manipulate information and achieve a preset goal, the TOL task requires subjects to apply many types of ability, such as complex visual and spatial planning, working memory and selective attention (21). Previous studies using fMRI have highlighted the various brain regions activated during the TOL task (21, 22). However, although many studies have used fMRI to assess executive functions during the TOL task, fMRI is expensive and not easy to move. In addition, many patients cannot adapt to a claustrophobic environment. Thus, it is necessary to explore a method which can assess executive functions and provide convenience for neurophysiologic studies and clinical diagnosis. In this study, we used NIRS to investigate hemodynamic responses to VFT and the TOL task in the prefrontal areas of patients with bipolar depression. We also hypothesized that NIRS can be used to assess cognitive abilities and may provide biomarkers for patients with bipolar disorder.

## Methods

### Participants

We recruited 43 patients with bipolar depression (17 males/26 females) diagnosed according to the DSM-V criteria from both the inpatient and outpatient populations of the Department of Psychiatry, Renmin Hospital of Wuhan University, from April 2017 to August 2017 and 32 healthy volunteers (15 males/17 females) to serve as healthy controls (HCs). All patients were right-handed and aged between 16 and 50. Subjects with neurological disease or other psychotic disorders, substance abuse, severe medical disease or cognitive dysfunction were excluded. The HCs also met the above criteria and had no family history of psychiatric disorders. Symptoms of mania and depression were evaluated in BD patients using the Hamilton Rating Scale for Depression (HAMD, 17-item) and the Young Mania Rating Scale (YMRS). All the patients had a 17-item HAMD score of >7 and a YMRS score of <10. Daily doses of all antipsychotics were converted to an equivalent dose of chlorpromazine; antidepressants, to that of imipramine; andanxiolytics / hypnotics, to that of diazepam (23). This study was approved by the ethics committees of Renmin Hospital of Wuhan University. Written informed consent was obtained from every participant and their parents (for minors).

### NIRS measurements

**Figure 1:**
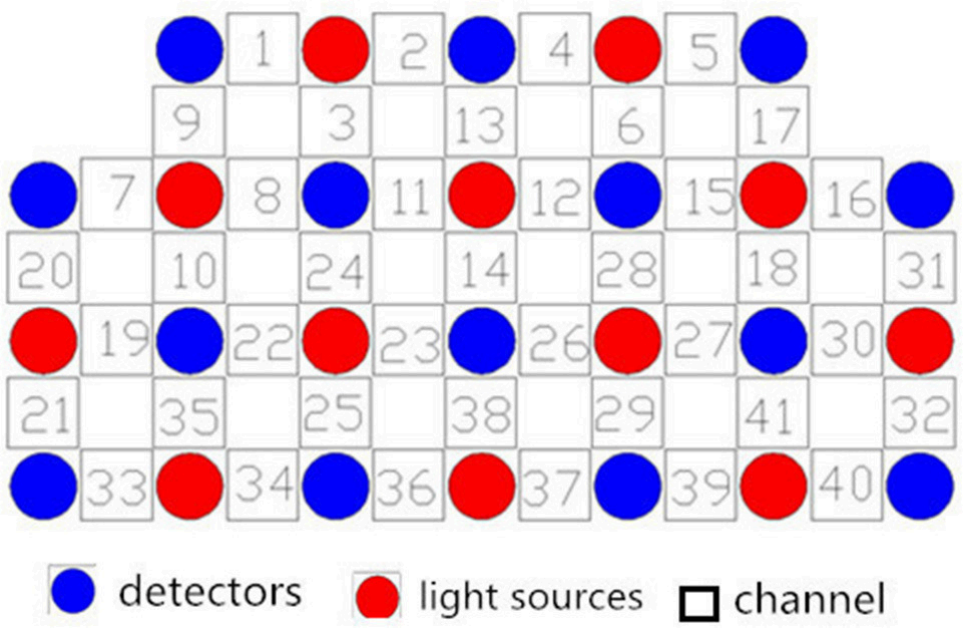
Locations of the channels of the near-infrared spectroscopy instrument. Probe positions illustrated in a 2D plane.

**Figure 2:**
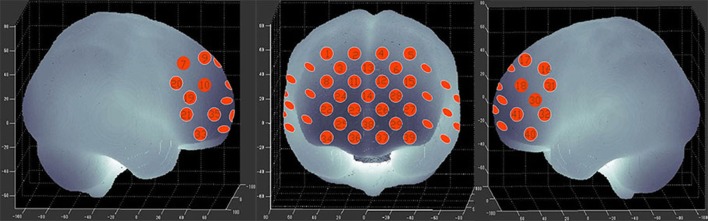
The 41 measuring positions of the NIRS device are superimposed on the 3D-reconstructed cerebral surface. The dimensional figures indicate the right temporal, frontal, and left temporal brain regions.

We used a 41-channel NIRS system (ETG-4000, Psyche-Ark Science & Technology Development Co., Beijing) to measure changes in the concentrations of oxygenated hemoglobin [oxy-Hb] and deoxyhemoglobin [deoxy-Hb] at two wavelengths (695 nm and 830 nm) of infrared light based on the modified Beer-Lambert law (24). The total oxygenated hemoglobin concentration is the sum of the [oxy-Hb] concentration and the [deoxy-Hb] concentration. We placed the source-detector probes on the participants' prefrontal areas as shown in Figures 1,2, and the positions of the probes were corroborated by many previous studies according to the International 10–20 system (25). The distance between a detector probe and injector probe pair was set at 3.0 cm, and the area between a detector probe and injector probe pair was defined as a “channel.” The sampling rate was set to 24 Hz. A 3D-magnetic space digitizer was used to record the 3 dimensional locations of NIRS probes on each participant's scalp. The corresponding location of each channel in the Montreal Neurological Institute (MNI) space were estimated by the probabilistic registration method (26).

### Activation task

#### VFT

Participants sat on a comfortable chair in front of the computer screen and were instructed to minimize any major body movements to avoid creating imaging artifacts. The task used was similar to that described by Ma et al. (27). This task completed needs for 4 min 30 s. At the beginning of the task, the screen has a 30 s blank period, and participants need to repeatedly count from one to five until task starts. Then a category name (e.g., fruits, vegetables, household appliances and four-legged animals) would appear on the computer screen, and participants needed to list as many items as possible that belong to the same category. After 30 s, the screen will return to blank, and participants would repeat counting procedure as it was done in the pre-task period. The task and post task block would appear alternatively, and each task block would require participant to name objects to a new category. We regarded the correct items generated during 4 task periods (fruits, vegetables, household appliances, and four-legged animals) as the task performance.

#### TOL

The TOL task required participants to provide an answer for the minimum number of steps required to move balls to a target position (28). Before the TOL task, the participants were introduced to and trained on the experimental rules by a trained psychiatrist, and then they were required to provide an answer for the minimum number of steps needed to move from picture A to picture B. At the beginning of the task, the screen has a 30 s blank period and participants can have a rest at this time. Then a picture would appear on the computer screen, and participants were required to give their answers by pressing number key of the keyboard, and each answer they gave was recorded by the computer. After pressing the key, the computer switched to the next picture automatically. During this 30 s task time, participants were required to answer as many question as possible. The task period last for 30 s, and followed by another task period after an interval of 30 s for rest. Unlike the VFT, during the rest periods of the TOL test, participants were not asked to say anything; they were instructed to sit in front of the computer in silence with their heads fixed and unmoving. To minimize the effects of occasional errors on the task, we repeated the test with 6 blocks. Each block consisted of a 30 s task period and 30 s rest period. The difficulty of the TOL questions were the same across blocks. The average rate of correct answers and the average time required to provide an answer were assessed during 6 task periods as the task performance. We defined the average rate of correct as the number of correct answers divided by the total number of answers.

### Statistical analyses

We calculated the mean [oxy-Hb] and [deoxy-Hb] changes during the task period in each channel for each participant; the [oxy-Hb] changes had a better signal-to-noise ratio than the [deoxy-Hb] changes (29). Increases in [oxy-Hb] can more directly reflect task-related cortical activation than decreases in [deoxy-Hb] (30). We used the NIRS-SPM to analyze the [oxy-Hb] data (31–33). First, we pre-processed the data by using the transfer function of hrf and a Wavelet-MDL detrending algorithm to remove noise and artifacts. We computed the mean and the standard deviation of each channel for each participant, and then converted the raw time course values to Z scores (34). We averaged all the blocks for each channel of the tasks to derive a grand averaged time course waveform of each channel. For each task, we calculated the average [oxy-Hb] in each block during the task and rest periods separately. In the first part of the rest period, the [oxy-Hb] changes may be affected by the task period, thus we regarded the last 5 s of the rest period as the baseline. The difference of the [oxy-Hb] changes between the task period and the last 5 s of the rest period were defined as the average [oxy-Hb] changes.

All statistical analyses were performed using SPSS version 19.0. Demographic characteristics such as age, duration of illness, years of education, TOL task and VFT performance between the bipolar depression group and the control group were compared with independent *t*-tests. Chi-squared tests were performed for gender-related items. The difference in age between two groups showed a trend level of significance (*p* < 0.088). In order to control the effect of age, we use the age as a covariate when comparing the mean [oxy-Hb] changes between two groups. The correlations between the mean [oxy-Hb] changes and the HAMD scores and task performance were analyzed by Partial correlation analysis and the age were used as a controlled variable. To assess laterality effects, we used repeated-measures ANOVA with channels and hemisphere (left vs. right) as two repeated measurements factors. Statistical significance was considered at *p* < 0.05 (two-tailed). Results were corrected for the number of channels by way of FDR correction (*p* < 0.05).

## Results

### Demographic characteristics and task performance

Table 1 summarizes the demographic characteristics and task performance of the two groups. There were no significant differences between patients with bipolar depression and HCs in gender, age, and education. Significant differences between patients with bipolar depression and HCs were observed in TOL task and VFT performance. Patients with bipolar depression had lower average rates of correct answers (*t* = −3.362, *p* = 0.001) and longer average answer times (*t* = 3.923, *p* < 0.000) than HCs in the TOL task. The differences between patients with bipolar depression and HCs were also statistically significant in the correct items generated during the vegetable and fruit blocks (*t* = −2.822, *p* = 0.006, and *t* = −2.687, *p* = 0.009) during the VFT. However, no significant differences were found in the correct items generated during the four-footed animal and family application blocks.

**Table 1:** Participants' demographic characteristics and task performance.

	**Bipolar depression mean ± SD**	**Healthy controls mean ± SD**	***P*-value**
**n**	**43**	**32**	**–**
Sex, male/ female, *n*	17/26	15/17	0.525
Age, year	26.7 ± 7.0	24.7 ± 2.4	0.088
Education, year	14.8 ± 2.4	15.5 ± 1.0	0.094
Duration of illness, year	3.5 ± 4.1	–	–
HAMD-17	20.3 ± 5.1	–	–
YMRS	2.4 ± 0.6		
**VFT PERFORMANCE**, ***n***			
Four-footed animal block	9.6 ± 3.3	9.9 ± 2.8	0.687
Vegetable block	9.9 ± 2.6	11.7 ± 2.9	0.006
Family application block	8.9 ± 2.7	9.8 ± 2.3	0.129
Fruit block	10.4 ± 2.6	12.0 ± 2.4	0.009
Average VFT performance	9.72 ± 2.15	10.81 ± 1.91	0.026
**TOL PERFORMANCE**, ***n***			
Average rate of correct responses,%	0.76 ± 0.23	0.91 ± 0.11	0.001
Average answer time, ms	10515.1 ± 3435.0	8031.4 ± 2010.9	<0.001
**MEDICATION**			
Lithium (mg/day)	430.0 ± 59.4	–	–
VPA (mg/day)	684.8 ± 188.0	–	–
Antipsychotics (mg/day)	125.0 ± 20.2	–	–
Antidepressants (mg/day)	91.9 ± 17.7	–	–
Anxiolytics (mg/day)	5.3 ± 2.6		

### Mean hemodynamic changes during the TOL task

**Figure 3:**
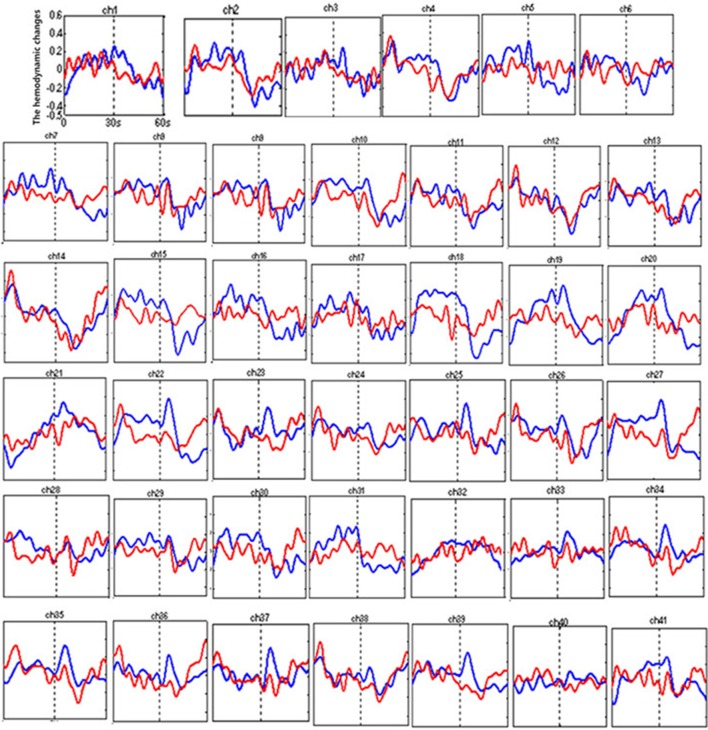
The time courses of the mean hemodynamic changes (*Z*-value) of 41 channels during the TOL task. The ordinate is the mean hemodynamic changes (*Z*-value), the abscissa is the time course of the task, the first part represents the task period, and the second part represents the rest period. Patients with bipolar depression (red); HCs (blue).

**Figure 4:**
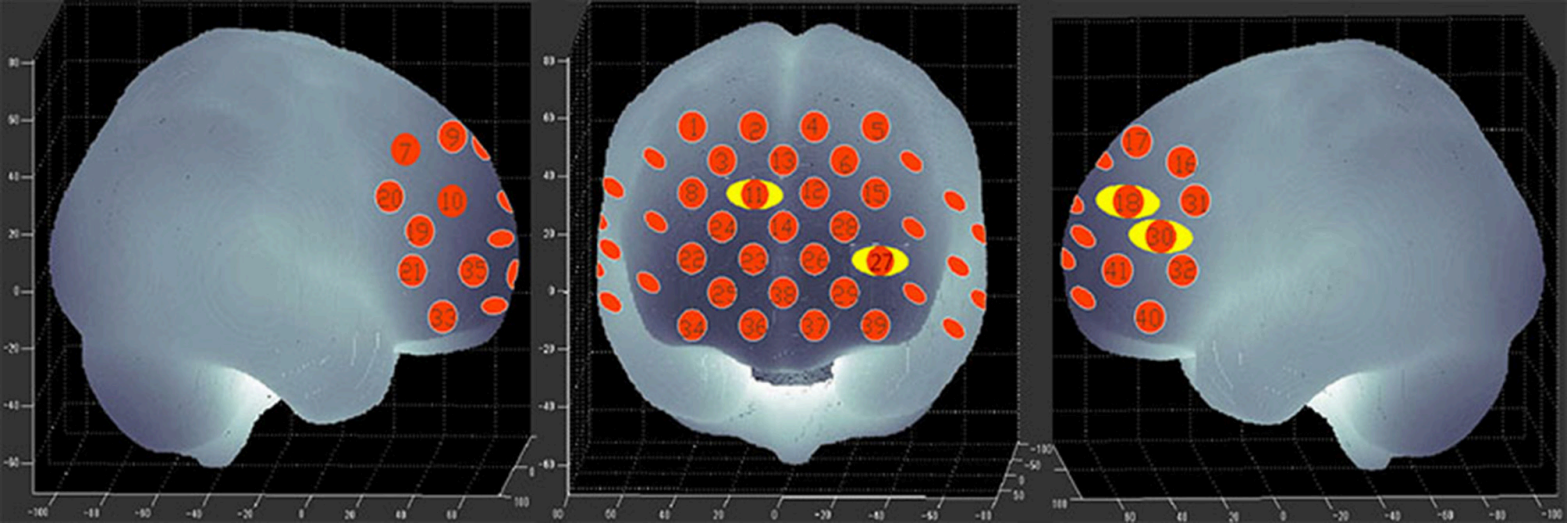
*P*-value significance map for mean hemodynamic changes during the TOL task. *P*-value significance map for mean hemodynamic changes in patients with bipolar depression compared with HCs during the TOL task. The yellow circles represent significantly smaller oxy-Hb changes than in the control group at the channels indicated.

Figure 3 shows the time courses of the mean hemodynamic changes in [oxy-Hb] signals during the TOL task between patients with bipolar depression and HCs. The difference of the mean [oxy-Hb] between the task period and rest period in patients with bipolar depression was smaller than the HCs in all four channels (ch11, ch18, ch27, ch30, *F* = 0.011–12.879, FDR *p* < 0.05, *p* = 0.041–0.049) during the TOL task. Figure 4 is the *P*-value significance map for the difference of the mean [oxy-Hb] between the task period and rest period in patients with bipolar depression compared with HCs during the TOL task.

### Mean hemodynamic changes during the VFT task

**Figure 5:**
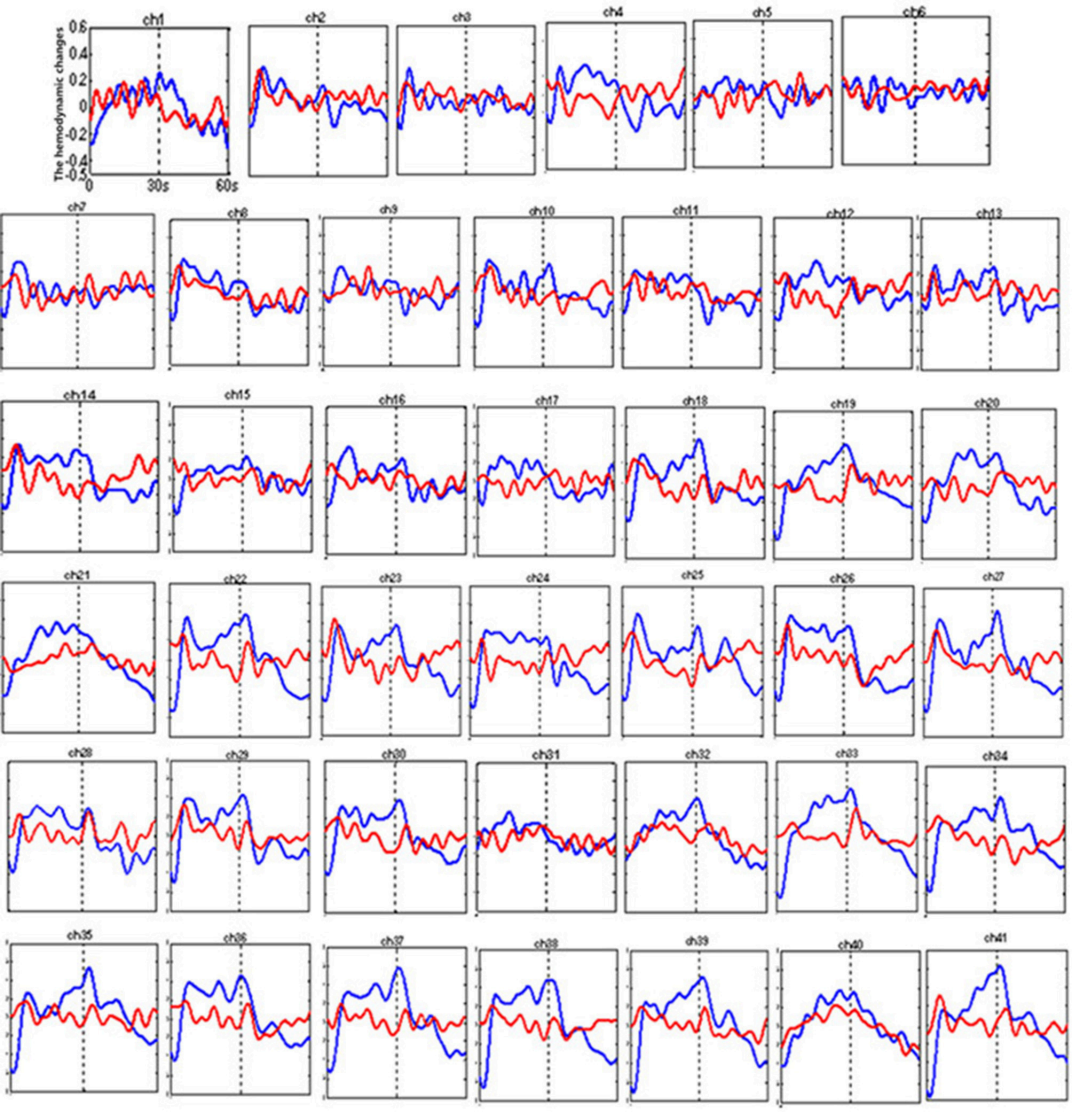
The time courses of the mean hemodynamic changes (*Z* value) of 41 channels during the VFT. The ordinate is the mean hemodynamic changes (*Z* value), the abscissa is the time course of the task, the first part represents the task period, and the second part represents the rest period. Patients with bipolar depression (red); HCs (blue).

**Figure 6:**
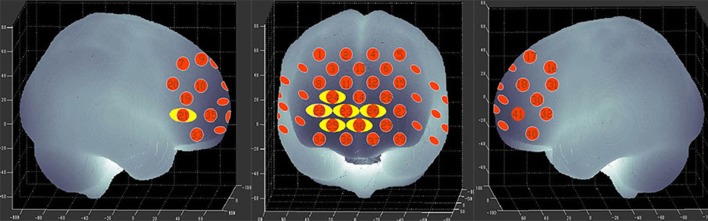
*P*-value significance map for mean hemodynamic changes during the VFT. *P*-value significance map for mean hemodynamic changes in patients with bipolar depression compared with HCs during the VFT. The yellow circles represent significantly smaller oxy-Hb changes than in the control group at the channels indicated.

Figure 5 shows the time courses of the mean hemodynamic changes in [oxy-Hb] signals during the VFT task between patients with bipolar depression and HCs. The difference of the mean [oxy-Hb] between the task period and rest period in patients with bipolar depression was significantly smaller than the HCs in all seven channels (ch21, ch22, ch23, ch24, ch25, ch26, ch38, *F* = 0.029–10.892, FDR *p* < 0.05, *p* = 0.027–0.046) during the VFT. Figure 6 is the *P*-value significance map for the difference of the mean [oxy-Hb] between the task period and rest period in patients with bipolar depression compared with HCs during the VFT.

### Correlation between NIRS data and clinical variables

**Figure 7:**
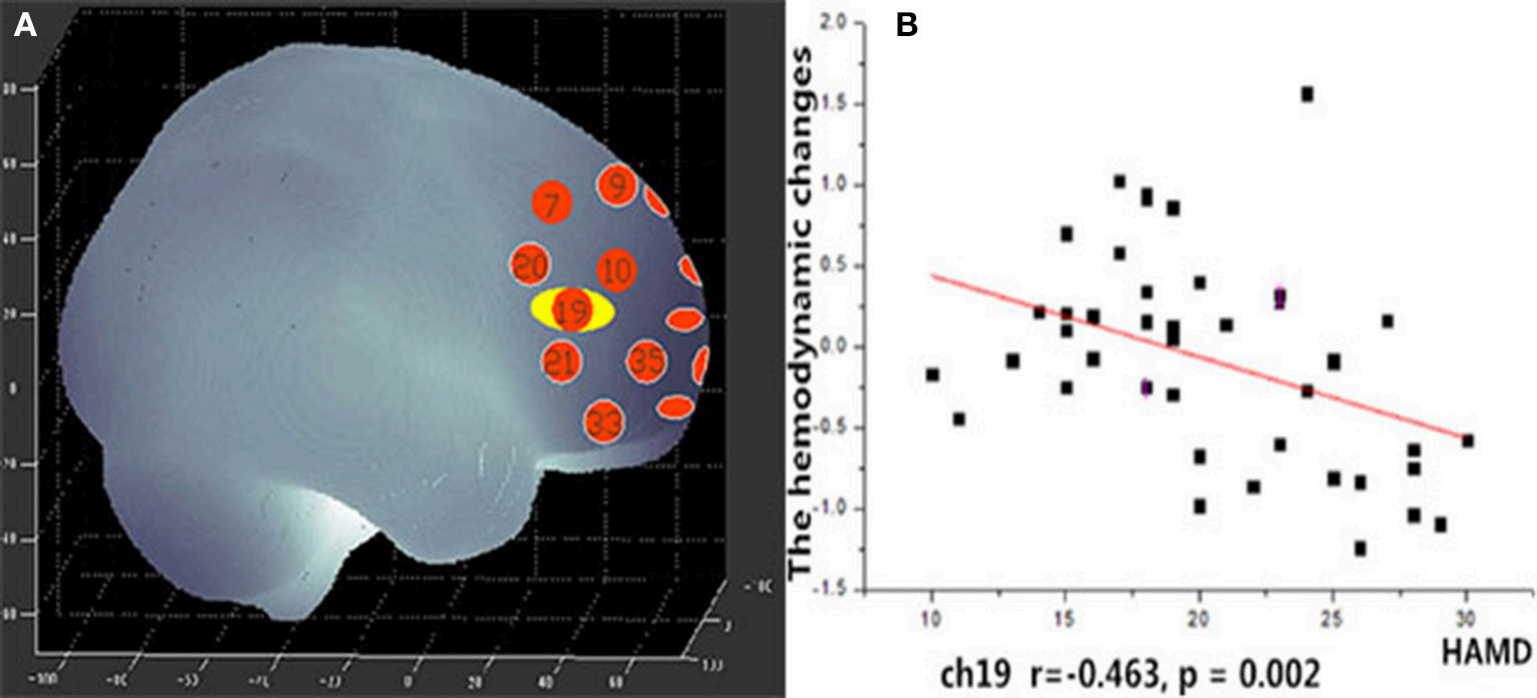
**(A)** Channel 19 shows a significant correlation between oxy-Hb changes and HAMD scores. **(B)** Scatter graph showing the relationship between HAMD scores and oxy-Hb activation in Channel 19.

During the VFT, a significant negative correlation was found between HAMD scores and the mean hemodynamic changes in the right dorsal-lateral prefrontal cortex (rDLPFC) in channel 19 (*r* = −0.460, *p* = 0.002; Figure 7). However, there were no significant correlations between HAMD scores and the mean hemodynamic changes in any channels during the TOL task. In addition, no significant correlations were found between the mean hemodynamic changes and other clinical variables such as years of education and YMRS scores in either patients with bipolar depression or HCs.

### Laterality analyses

The repeated-measures ANOVA for VFT revealed significant main effects of group [*F* = 9.771, *p* = 0.003], hemisphere [*F* = 5.835, *p* = 0.018], channel [*F* = 2.332, *p* = 0.002], group × hemisphere [*F* = 6.740, *p* = 0.011] and significant group × channel interaction [*F* = 1.746, *p* = 0.030]. The mean [oxy-Hb] change was significantly smaller over the left relative to the right region. While the repeated-measures ANOVA for TOL task revealed significant main effects of group [*F* = 8.376, *p* = 0.005], but no significant main effect of hemisphere, channel, and no significant group × hemisphere and group × channel interaction.

## Discussion

### Reduced DLPFC activation in patients with bipolar depression during the TOL task

To the best of our knowledge, this is the first study to evaluate brain activation in patients with bipolar depression during the TOL task by measuring hemodynamic changes using NIRS. The present data indicate that patients with bipolar depression had lower activation levels during the TOL task in the bilateral dorsal-lateral prefrontal cortex (DLPFC) in channels 11, 18, 27, and 30 (Figure 4) than the HCs. Our study confirmed the only previous study of executive function during the TOL task with NIRS, which indicated that the DLPFC is crucial for planning and problem-solving abilities (22). In addition, this result is also consistent with several fMRI studies that show that the prefrontal cortex (PFC) is reliably activated, including its inferior, dorsolateral, and anterior aspects, during planning tasks (35, 36). However, the results obtained by Rive et al. (37) indicate increased frontostriatal activity in unmedicated bipolar depression patients compared to MDD patients and HCs. As no previous studies report hemodynamic changes during the TOL task with NIRS, more studies are needed to validate these results.

### Reduced PFC/rVLPFC/rDLPFC activation in patients with bipolar depression during the VFT

The VFT has been commonly used as an activation task with NIRS. In this study, we found that patients with bipolar depression had lower activation levels during the VFT in both the right and left PFC in channels 25, 38, 23, 26, and 24 (Figure 6) than the HCs. Moreover, less activation was observed in the right ventrolateral prefrontal cortex (VLPFC) in channel 21 and the right DLPFC in channel 22 during the VFT than in the HCs. However, the activation of various brain areas during the VFT in previous studies was reported inconsistently. Most studies of the VFT showed decreased activation in patients with bipolar disorder compared with HCs (12, 17, 38). However, Kubota et al. reported increased activation, and Kameyama et al. found the activation to change over time in patients with bipolar disorder compared with HCs during the VFT (39, 40). The differences in these results may be related to the time course of the task; for example, we used 60 s for each initial syllable, while in Takizawa's study, the time interval used was 20 s (12).

### The poor task performance of patients with bipolar depression

In the TOL task, the average rate of correct answers and the average answer time, respectively, reflect the accuracy and efficiency of planning and problem-solving ability, which is essential for everyday life. Our study demonstrated that patients with bipolar depression performed poorly and needed more time to complete the task. In the VFT, the number of words generated during the fruit, vegetable blocks and the average VFT performance were significantly lower in patients with bipolar depression than in the HCs. These results were consistent with a previous report that found that the ability to generate words is associated with frontal lobe function (41). Thus, the prefrontal cortex is essential for executive functions.

### Regional brain activation and HAMD scores

Unlike the TOL task, the mean hemodynamic changes in the right DLPFC were found to be negatively correlated with HAMD scores during the VFT. Ono et al. (42) reported that activation in the right temporal gyrus is correlated with HAMD scores during the Iowa Gambling task, but no correlation was found during the VFT. Nishimura et al. (17) found that hypomanic symptom severity was correlated with activation in the left DLPFC. Noda et al. (43) reported the mean increase in oxy-Hb during the VFT in the frontal and right temporal cortex showed a significant negative correlation with the total score of the HAMD 21-item version in patients with MDD. This suggests that the symptoms of bipolar disorder may have some relation to the dysfunction of the DLPFC. In this study, we also found significant main effects of hemisphere and group × hemisphere interaction, the mean [oxy-Hb] change in the left region was significantly smaller than the right region. In recent years, many imaging studies have focused their attention on Laterality effects of brain. Okada et al. (44) found depressed patients had poor performance and the left PFC showed reduced activity than controls during a VFT of fMRI study. This may suggest that the left hemisphere of the brain is more involved in language processing than the right hemisphere.

### Limitations

This study has several limitations. First, NIRS can only measure cortical regions rather than the deep structures of the brain. Second, the sample size used was relatively small. Third, all of the patients in our study were taking medications at the time of measurement, medication effects may be present and cannot be easily controlled (45). As far as we know, no clear evidence of the effects of medications on NIRS signals has been demonstrated. Finally, our study is a cross-sectional study, and we only evaluated patients with bipolar depression.

## Conclusions

Our study demonstrated that planning and problem-solving abilities are associated with the DLPFC and that patients with bipolar depression demonstrated hypoactivity in this area. Patients with bipolar depression exhibited dysfunctional regions in the PFC, the right VLPFC (channel 21) and the right DLPFC (channel 22), which were related to executive function. Moreover, the TOL task with NIRS can be used to in clinical to assess planning and problem solving abilities of bipolar patients, which are essential for daily life in bipolar disorder patients. In addition, the finding that group differences in cortical activity were primarily over right frontal regions is interesting and need more studies to explain it. In a future study, we will recruit more participants in different mood states for a longitudinal assessment.

## Author contributions

LF and ZL conceived and designed the study. LF, ZL, JX, and DX performed the experiments. LF analyzed experimental results. LY, YW, and LX assisted with data analyze. LF wrote the paper. LF, ZL, HW, and GW reviewed and edited the manuscript. All authors read and approved the manuscript.

### Conflict of interest statement

The authors declare that the research was conducted in the absence of any commercial or financial relationships that could be construed as a potential conflict of interest.
